# Increasing lifetime exposure to extreme fire weather under climate change in Europe

**DOI:** 10.1088/1748-9326/ae93de

**Published:** 2026-09-21

**Authors:** R Pietroiusti, J Hetzer, M Turco, A Laridon, Q Lejeune, S Prudencio Montaño, D Paprotny, W Thiery

**Affiliations:** 1 Vrije Universiteit Brussel, Department of Water and Climate, Brussels, Belgium; 2 Senckenberg Biodiversity and Climate Research Centre (SBiK-F), Frankfurt am Main, Germany; 3 Department of Physics, Regional Campus of International Excellence (CEIR) Campus Mare Nostrum, University of Murcia, Murcia, Spain; 4 Institute of Marine and Environmental Sciences, University of Szczecin, Szczecin, Poland; 5 Potsdam Institute for Climate Impact Research (PIK), Member of the Leibniz Association, Potsdam, Germany; 6 Baltic Climate Centre, University of Szczecin, Szczecin, Poland

**Keywords:** fire weather, fire weather index, lifetime exposure, projections, Europe, Portugal

## Abstract

Climate change increases fire weather globally. Hot, dry and windy conditions raise the likelihood of fires igniting and spreading and make suppression more challenging. With further warming, fire weather is projected to intensify across Europe, yet implications for today’s young generations remain unclear. Here, we analyse lifetime exposure to extreme fire weather across Europe using an ensemble of bias-adjusted and downscaled global climate models. To this end, we developed *dem4cli*, an open-source Python package that integrates demographic data into climate hazard assessments. We find younger generations are projected to be disproportionately exposed compared to older generations across all warming pathways, while also benefitting most from ambitious mitigation. Across Europe, individuals born in 2025 are projected to experience, on average, 3.2 additional years of exposure to extreme fire weather compared to those born in 1950, corresponding to an 84% increase, with larger increases observed in Southern and Eastern Europe. Focusing on Portugal, under current policies, people born in 2025 are projected to experience nearly twice the exposure compared with those born in 1950, and up to 2.5 times more in the most exposed regions of northeastern Portugal. Under a 1.5$^ {\circ}$ C pathway, lifetime exposure is substantially reduced. Each additional degree of global warming by 2100 adds approximately 270 days of extreme fire weather to the lifetime exposure of a person born in Portugal in 2025. These results reveal pronounced intergenerational inequalities in exposure to fire weather and underscore the urgency of ambitious mitigation to limit cumulative exposure for younger generations.

## 1. Introduction

Climate change is driving increases in fire weather across the world [1–4]. Hotter and drier conditions dry fuels, increasing the likelihood of ignition and enabling faster fire spread that is more difficult to suppress [5].

Across Europe, wildfires pose a risk to populations. In the summer of 2025, more than 1 Mha burned in the European Union, breaking records since 2006, when satellite-based estimates from the European Forest Fire Information Service (EFFIS) began. Fires particularly affected Spain, Portugal [6, 7] and Southeast Europe [8], causing dozens of casualties and hundreds of injuries. Previous active fire seasons occurred in 2017, which recorded the second-highest burned area after 2025, and in 2021–2023 [9–12], with highly impactful fires affecting Greece [13], Portugal [14, 15] and Turkey [13]. Most of the wildfire activity in Europe is concentrated in the Mediterranean, which accounts for the majority of burned area in Europe [16, 17] and is among the most fire-affected regions worldwide [3].

Within this region, Portugal stands out as the European country most severely affected by wildfires, with over 100 000 ha burned annually on average in the past two decades and the highest proportion of national territory burned each year [18]. Portugal is home to some of the most fire-prone regions in Europe [19]. Severe wildfire events in 2017, 2024 and 2025 caused multiple fatalities, and injured dozens to hundreds of people [6, 14, 15, 20].

Fire activity emerges from the interplay between vegetation characteristics, climatic variability, and human management. Nonetheless, studies have shown that climatic conditions exercise a dominant influence on the interannual variability of fire activity in many parts of the world, including the Mediterranean [3, 21–23]. Fire weather is projected to increase across Europe with further warming, and to expand into areas previously rarely exposed to high levels of fire danger [24, 25]. In the Mediterranean, climatic conditions are projected to become hotter and drier, with warming amplified during summer and precipitation declines projected in all seasons [26–28]. Such changes would increase fire activity in an already fire-prone region [29], recognized as a climate change hotspot [26, 30, 31].

While future increases in fire danger across Europe are well established, the intergenerational implications of this have not been explored. Today’s children have little responsibility for past emissions, yet they will be disproportionately exposed to the impacts of climate change over their lifetimes and will bear much of the responsibility for mitigation efforts to limit future warming and associated risks [32, 33]. Although cohort-based approaches have recently been developed to quantify intergenerational differences in lifetime exposure to climate extremes [34, 35], such assessments, and what they imply for intergenerational equity, are rarely incorporated into considerations on the relative desirability of different mitigation or policy pathways [36, 37], despite their relevance for evaluating the long-term societal implications of climate policy.

Here, we assess how exposure to extreme fire weather may differ across the lifetimes of different generations. Fire weather is represented using the Canadian Fire Weather Index (FWI) and examined under stylized, policy-relevant global warming pathways reaching 1.5$^ {\circ}$ C–3.5$^ {\circ}$ C by 2100. We use these pathways to emulate regional fire weather responses to global warming based on input state-of-the-art global climate model (GCM) simulations from six models and four scenarios (SSP126, SSP245, SSP370, SSP585), statistically downscaled at 0.1$^ {\circ}$ resolution, and combine this with data on population density and life expectancy.

We build on previous studies estimating intergenerational inequity in exposure to climate extremes [34, 35]. Our analysis extends this work by estimating exposure at subnational level, updating the demographic data, and improving the emulation of regional climate responses under stylized warming pathways. We integrate these developments into *dem4cli* (‘demographics for climate’), a flexible open-source Python package for estimating age-specific and lifetime exposure to any climate hazard.

In section 2 we describe our data and methods, and in section 3 we present results for Europe and then focus on Portugal, given its relevance as one of Europe’s most fire-prone countries.

## 2. Methods

### 2.1. Fire weather data and definition of extreme fire weather days

To study fire-prone weather conditions, we used the FWI, a dimensionless measure of meteorological fire danger that integrates temperature, humidity, wind speed, and precipitation, and accounts for antecedent conditions influencing fuel dryness and flammability [38–41]. We obtained daily FWI values from [24], calculated from six CMIP6 GCMs under historical forcings and four Shared Socioeconomic Pathway (SSP-RCP) scenarios for 1950–2100 (table A1). Input variables were statistically downscaled and bias-adjusted against ERA5-Land over 1985–2014. We also used ERA5-Land FWI for the period 1985–2014 from [24] to compare reanalysis and model trends. All data were bilinearly interpolated to a regular 0.1$^ {\circ}$ grid to match the gridded population data.

We defined ‘extreme fire weather days’ as days exceeding the local 95th percentile of FWI, estimated over the 1985–2014 reference period (FWI95d). This period was selected to match the bias-adjustment reference period and ensure consistency across climate model simulations and the reanalysis reference data. We followed previous studies in using a local percentile-based definition of extreme fire weather [1, 2, 42]. By construction, this definition corresponds to approximately 18 extreme fire weather days occurring per year during the reference period (figures S1 and S2).

We additionally present results based on two absolute FWI threshold classes used by the EFFIS fire danger forecast service to indicate ‘very high’ fire danger (38 ${\unicode{x2A7D}}$ FWI < 50) and ‘extreme’ fire danger (FWI $\unicode{x2A7E}$ 50) [43]. Although operational fire danger thresholds may vary across regions depending on local climate, fire regimes, vegetation and management practices, we use the EFFIS thresholds as a common reference scale for comparison across Europe. Relative indicators enable consistent comparisons across heterogeneous climate zones by normalizing for local climatology, while absolute thresholds foreground regional contrasts in baseline climate, identifying areas that are hotter and drier in absolute terms. Both types of indicators are relevant for assessing meteorological fire danger. Due to its rarity, we include the ‘very extreme’ fire danger class, introduced in 2021 (FWI${\unicode{x2A7E}}$70), within the ‘extreme’ danger class.

### 2.2. Dem4cli: a flexible package to estimate age-specific and lifetime exposure to climate hazards

**Figure A1.**
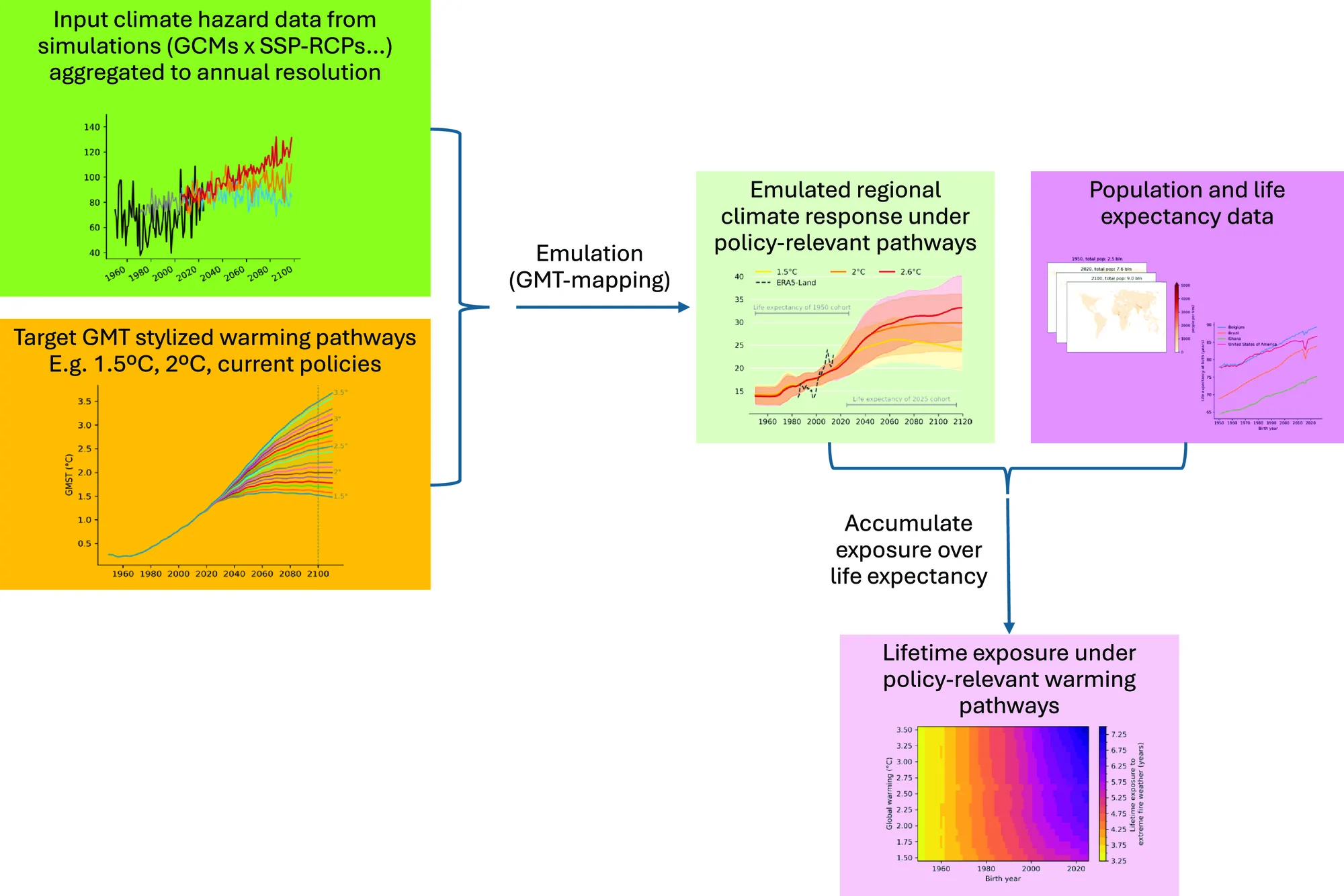
Lifetime exposure methodology available within *dem4cli*. User-provided climate hazard simulationsaggregated to annual resolution are provided as input (green) and are remapped using a GMT-mapping approach to emulate the climate response under user-defined stylized warming trajectories (yellow). In this study we create the stylized trajectories by combining historical multi-dataset GMT time series from AR6 with projections from the AR6 Scenario Explorer projections, to construct 21 stylized pathways that in 2100 reach GMT anomalies ranging from 1.5$^ {\circ}$ C to 3.5$^ {\circ}$ C at regular intervals of 0.1$^ {\circ}$ C. The GMT-mapping procedure results in emulated regional climate responses under the target warming pathways (light green), which can then be combined with data on population density and lifetime exposure (purple) to calculate lifetime exposure across regions (e.g. countries or sub-national units) for each birth year (light purple). See Methods and supplementary text S1.

We present *dem4cli*, a new Python package that can be used to integrate demographic and climate data to estimate age-specific and lifetime exposure to climate hazards, building on previous studies [34, 35, 44]. Lifetime exposure is defined as the cumulative number of hazard occurrences experienced by individuals born in a given year and location over their lifetime (figure A1). The package allows analyses at national and subnational scales, supports user-provided climate or impact model ensembles, and allows for using high-resolution climate data.

The package allows users to provide their own climate hazard or impact simulations. Ancillary package data includes gridded population data, country and administrative boundaries, and data on age group distributions and life expectancy (section 2.3.3), which can be used to estimate gridscale age-specific exposure at time windows or warming levels, or lifetime exposure. Instead of carrying out lifetime exposure analyses along the original SSP-RCP pathways of the input simulations, *dem4cli* can be used to apply an emulation approach, whereby, based on target stylized global mean temperature (GMT) trajectories (section 2.3.1), input simulations are remapped by matching their GMTs with those of the target to emulate the regional climate response to warming (section 2.3.2). In this way, GCMs with different climate sensitivities can be compared and results can be communicated based on policy-relevant warming pathways [45].

Compared to earlier studies, in *dem4cli* we update population and demographic datasets, improve consistency in the stylized warming pathways between historical and projected warming by basing both on the latest AR6 assessment, improve the emulation approach to better isolate the forced signal of change, and add flexibility for user-defined analyses.

Section 2.3 describes the specific application of *dem4cli* for this study, while the full range of options available for users are provided in the supplementary text S1.

### 2.3. Estimating lifetime exposure to extreme fire weather days

Using *dem4cli*, we estimated lifetime exposure to extreme fire weather for individuals born between 1950 and 2025 in Europe under stylized warming trajectories reaching 1.5$^ {\circ}$ C–3.5$^ {\circ}$ C by 2100.

#### 2.3.1. Construction of stylized global warming pathways

We constructed 21 stylized GMT pathways reaching 1.5–3.5$^ {\circ}$ C in 2100 at regular 0.1$^ {\circ}$ C intervals (figure A1). Following [35], we designed these by linearly interpolating global warming pathways obtained from the IPCC AR6 Scenario Explorer [46] for 2000–2100. These were extended backward using AR6-assessed GMST observations for 1950–1999 [27, 36, 47], smoothed with a 21-year moving average to remove natural variability, and extended beyond 2100 using linear extrapolation of the final decade.

The 1.5$^ {\circ}$ C warming pathway we thus constructed corresponds to a low-overshoot pathway that limits warming to 1.5$^ {\circ}$ C in 2100 after a peak of approximately 1.6$^ {\circ}$ C in the 2060 s. This pathway corresponds to the most ambitious category of mitigation pathways assessed in the IPCC AR6 (i.e. C1), which limit warming to 1.5$^ {\circ}$ C with no or limited overshoot [36, 48].

#### 2.3.2. Remapping input simulations to emulate regional impacts along stylized warming pathways

We emulated regional fire weather responses using a GMT-mapping approach, following [34, 35]. Such approaches assume that the local forced response of a climate indicator is primarily determined by the level of global warming [49, 50].

For each input GCM simulation from the SSP-RCP experiments (table A1), we derived annual grid-cell counts of extreme fire weather days together with the corresponding modelled annual GMT anomaly timeseries. We then smoothed both quantities using a 21-year moving average to isolate the forced response. Since the FWI data were bias-adjusted against 1985–2014, we rebased each model’s GMT timeseries so that mean warming over this period matched ERA5 warming relative to 1850–1900 (0.74$^ {\circ}$ C).

Then, for each target warming trajectory and input simulation, we identified the simulation year whose GMT anomaly most closely matched the target GMT anomaly in each year. The resulting year-to-year mapping was used as a remapping recipe, linking each target year to a corresponding year in the original simulation. We then applied this mapping to the gridded fire weather fields to emulate regional patterns of extreme fire weather along each target warming trajectory (figure S3).

Simulations were only used if the absolute GMT difference never exceeded 0.2$^ {\circ}$ C, ensuring sufficient consistency between input and target warming levels. Consequently, lower-warming trajectories (e.g. 1.5$^ {\circ}$ C) are informed by larger model ensembles than higher-warming trajectories (table A2).

#### 2.3.3. Population and administrative units data

We used gridded population data from the COMPASS project [51], updated in this study to align with the latest SSP v3.2 projections [52–54], at 0.1$^ {\circ}$ resolution. These data are derived by spatially disaggregating national population estimates from the United Nations World Population Prospects (UNWPP) [55] and SSP projections. Life expectancy data was taken from UNWPP2024. Following previous studies [34, 35], we estimated life expectancy at birth conditional on survival to age 5 and converted period life expectancy to cohort life expectancy, accounting for improvements in life expectancy, by adding 6 years, based on [56].

We conducted the analysis at national, NUTS2, and NUTS3 levels using the 2016 European NUTS classification [57]. A total of 1440 NUTS3 and 313 NUTS2 regions were included after excluding regions too small to be resolved at the resolution of the climate data. For Kosovo and Bosnia and Herzegovina, where NUTS classifications are unavailable, only national-level results are provided. Results were aggregated to IPCC AR6 regions based on centroid location.

#### 2.3.4. Estimating lifetime exposure

For each region and year, exposure was calculated as the population-weighted average number of extreme fire weather days, thus accounting for the spatial distribution of the population within each region. Lifetime exposure was then obtained by summing annual exposure from birth to death, including fractional exposure in the final year, and expressed as the total time exposed and the fraction of lifetime exposed. Additionally, we estimated the increase in exposure per degree of warming by fitting an ordinary least squares regression to ensemble mean results of lifetime exposure, separately for different cohorts and regions. Finally, we decomposed the difference in lifetime exposure between younger and older generations into contributions from climate change-driven increases in fire weather and from increases in life expectancy. To do so, we estimated lifetime exposure for each birth cohort under a counterfactual scenario in which annual fire weather exposure was held constant at its average 1950–1970 level to estimate the fraction due to increasing life expectancy, with the remaining fraction due to climate-driven increases in fire weather. We present results as ensemble means and present sensitivity to this choice in figure S7.

This framework enables comparisons of cumulative lifetime exposure across birth cohorts, regions, and warming pathways. The emulation approach (section 2.3.2) allows us to quantify changes in exposure per degree of warming and across policy-relevant warming scenarios. The 1.5–3.5$^ {\circ}$ C range reflects plausible future warming outcomes under a range of policy and climate sensitivity scenarios [58, 59], while ensuring sufficient simulations are available for robust emulation.

## 3. Results

### 3.1. Increasing lifetime exposure to extreme fire weather in Europe

**Figure 1.**
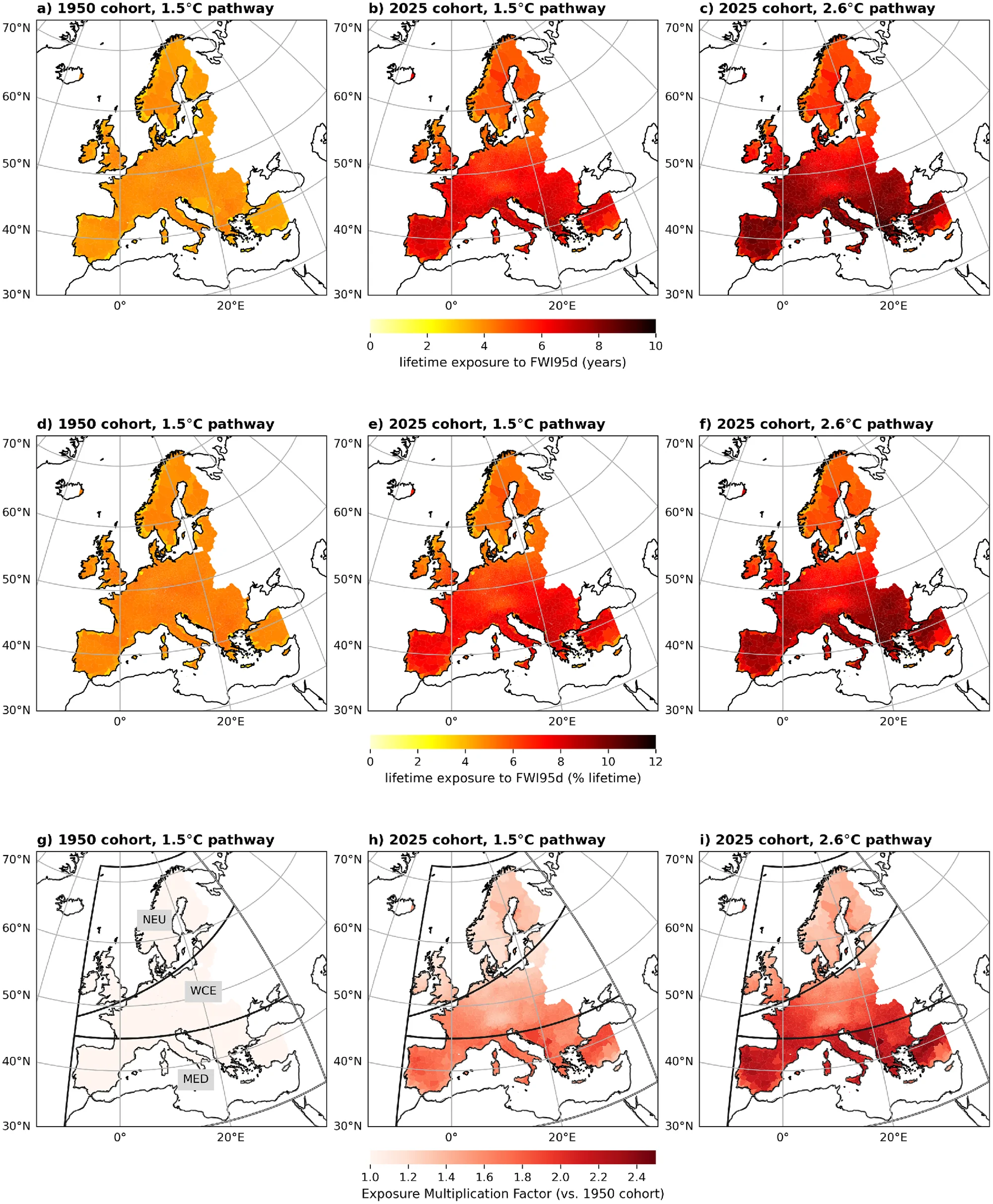
Lifetime exposure to extreme fire weather days (FWI95d) in Europe, at NUTS3 level. Number of years exposed (a)–(c), percentage lifetime exposed (d)–(f) and exposure multiplication factors relative to the 1950 birth year (g)–(i), for people born in 1950 (a), (d), (g) and 2025 (b), (c), (e), (f), (h), (i) under 1.5$^ {\circ}$ C (a), (b), (d), (e) and 2.6$^ {\circ}$ C (c) and (f) warming pathways. Multi-model mean is shown. See figures S4 and S5 for the results at the country and NUTS2 level. Results for all countries, NUTS2 and NUTS3 regions are included in tabular format as Supplementary Data.

We projected lifetime exposure to extreme fire weather in Europe at national and sub-national NUTS2 and NUTS3 levels under different warming pathways (figures 1, S4 and S5). Based on these projections, we find lifetime exposure would increase for younger birth cohorts and with higher levels global warming (figure 1), with the largest increases in Southern and Eastern Europe, and parts of Western and Central Europe.

We find little variation in the lifetime exposure of people born in 1950 across warming scenarios. At the NUTS3 level, we find they would be exposed to 0.5–4.3 years (1%–6% of lifetime, multi-model mean, range across regions) of extreme fire weather regardless of the warming pathway (figures 1(a) and (d)). For the 2025 birth year, projected exposure varies strongly across regions and scenarios. Under a pathway that reaches 1.5$^ {\circ}$ C in 2100, lifetime exposure would range from 0.6–6 years (1%–7% of lifetime) in Northern Europe, 2.4–7.2 years (3%–8% of lifetime) in Western and Central Europe, and 1.5–7.7 years (2%–9% of lifetime) in Southern Europe. In a 2.6$^ {\circ}$ C scenario, corresponding to the median expected end-of-century warming under current policies in the latest assessment of the United Nations Emissions Gap Report [58], projected exposure would increase to 0.7–7.4 years (1%–8% of lifetime) in Northern Europe, 2.9–8.6 years (3%–10% of lifetime) in Western and Central Europe, and 1.6–9.5 years (2%–11% of lifetime) in Southern Europe (figures 1(c) and (f)).

We find young generations are projected to experience substantially more extreme fire weather than older ones. Across NUTS3 regions in Europe, individuals born in 2025 would face on average 3.2 additional years of exposure compared to those born in 1950 under current policies (2.5–3.9, multi-model mean ± 1 SD), corresponding to an average increase in exposure by 84% (69%–99%, figure 1(i)). In some regions in Southern Europe, differences between generations would exceed five years, corresponding to an increase by a factor of up to 2.5 (2.1–2.9, figure 1(i). The largest increases are projected in regions of Turkey, Italy, southwestern France, the Iberian Peninsula, Greece, and the Balkans (figure 1(i)).

We decomposed the difference in lifetime exposure between the 1950 and 2025 birth cohorts into the contributions of increasing life expectancy and climate change-driven increases in extreme fire weather. Under a current policies pathway, we find that, on average across Europe, 86% (84%–87%) of the difference is due to climate change-driven increases in fire weather, whereas the remaining 14% (13%–16%) is due to longer lifetimes. In the most exposed Southern European countries the effect of climate change is larger, explaining up to over 90% of the difference in lifetime exposure between the cohorts (figure S6).

Aligning current policies with the 1.5$^ {\circ}$ C Paris Agreement target would reduce exposure in all regions (figure 1). Exposure for the 2025 birth cohort would decrease by 1.3 (0.6–2) years on average across Europe, 1 (0.2–1.9) years in Northern Europe, 1.3 (0.3–2.3) years in Western and Central Europe, 1.6 (1.0–2.1) years in Southern Europe, with reductions exceeding 2 years in the most affected regions in Southern Europe.

**Figure A2.**
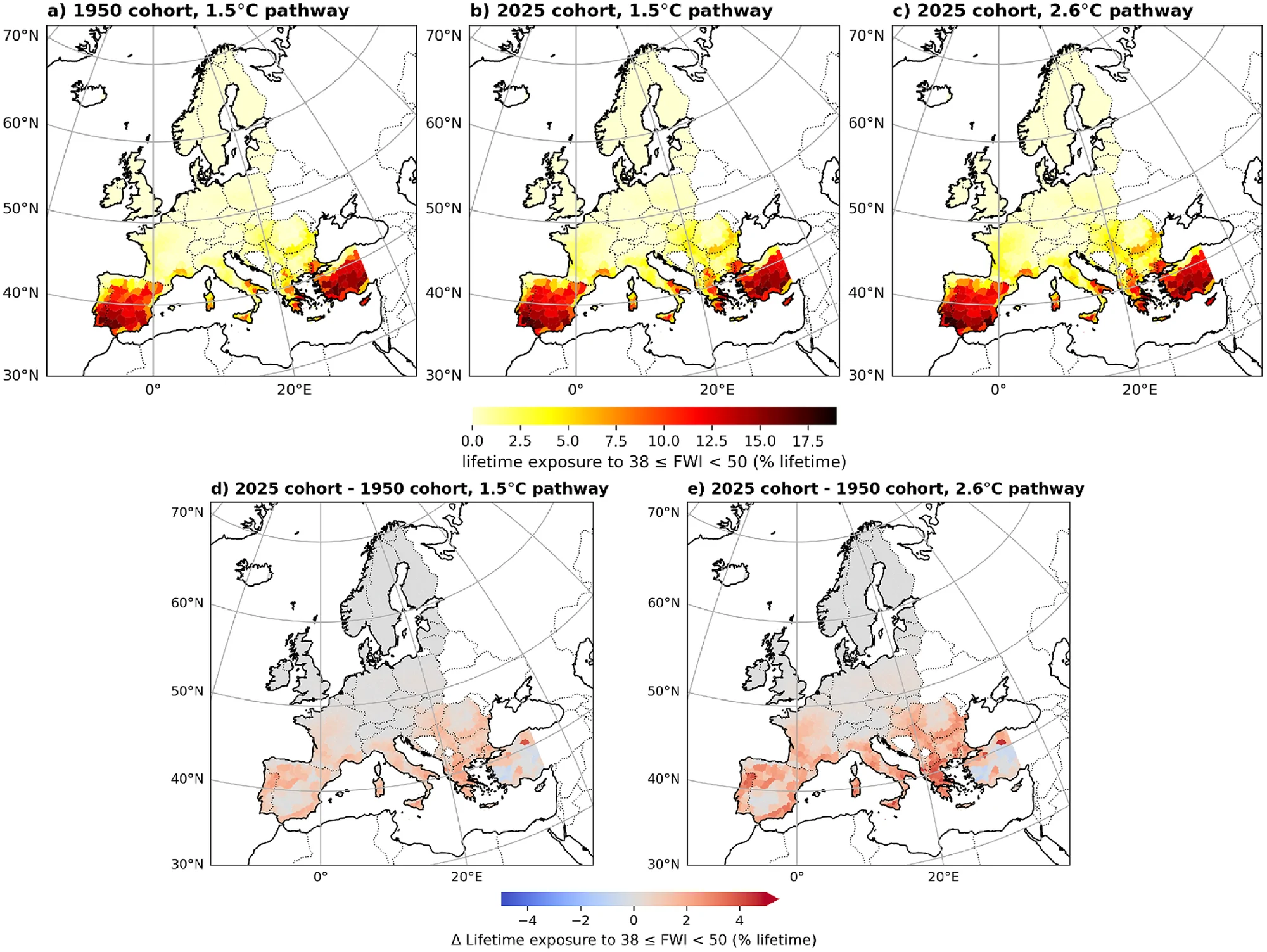
Lifetime exposure to very high fire danger days based on an absolute magnitude EFFIS threshold (38 $\unicode{x2A7D}$ FWI < 50) in Europe, at NUTS3 level. Percentage lifetime exposed (a)–(c) for people born in 1950 (a) and 2025 (b) and (c) under 1.5$^ {\circ}$ C (a) and (b) and 2.6$^ {\circ}$ C (c) warming pathways. Change in percentage of lifetime exposed to very high fire danger days between 1950 cohort and 2025 cohort under 1.5$^ {\circ}$ C (d) and 2.6$^ {\circ}$ C (e) warming pathways. Multi-model mean is shown.

When considering definitions of fire danger using the absolute magnitude thresholds adopted by EFFIS for fire danger forecasts, we find exposure to very high fire danger (38 $\unicode{x2A7D}$ FWI < 50) is projected to be concentrated in Southern Europe, particularly in the Iberian Peninsula, Turkey, Greece, Italy, Southern France and Eastern Europe (figures A2(a)–(c)). The strongest increases in lifetime exposure are projected in Iberia, Greece, Italy, Western France and Eastern Europe, (figures A2(d) and (e)). In these regions, people born in 2025 would experience up to 4 percentage points more of their lifetime exposed to very high fire danger than those born in 1950 under a 1.5$^ {\circ}$ C pathway, and up to 5 percentage points more under a 2.6$^ {\circ}$ C pathway.

**Figure A3.**
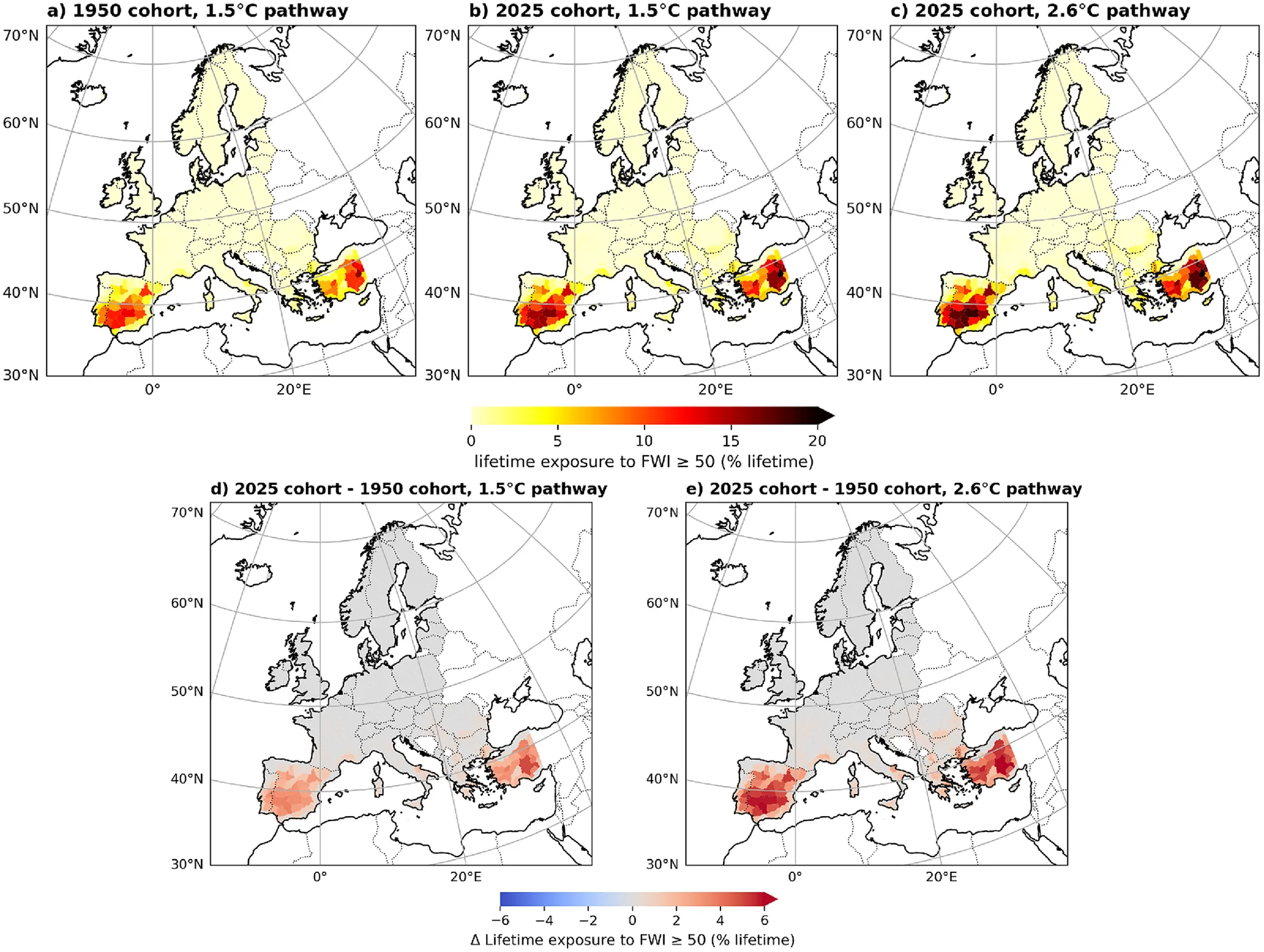
Lifetime exposure to extreme fire danger days based on an absolute magnitude EFFIS threshold (FWI $\unicode{x2A7E}$ 50) in Europe, at NUTS3 level. Percentage lifetime exposed (a)–(c) for people born in 1950 (a) and 2025 (b) and (c) under 1.5$^ {\circ}$ C (a) and (b) and 2.6$^ {\circ}$ C (c) warming pathways. Change in percentage of lifetime exposed to very high fire danger days between 1950 cohort and 2025 cohort under 1.5$^ {\circ}$ C (d) and 2.6$^ {\circ}$ C (e) warming pathways. Multi-model mean is shown.

Although exposure to extreme fire danger (FWI $\unicode{x2A7E}$ 50) is projected to be spatially more limited than very high fire danger, in location where it occurs, its frequency is projected to increase substantially. Under current policies, in the most affected areas, people born in 2025 would experience up to 7 additional years of lifetime exposure compared with those born in 1950 (6–7 additional percentage points of the lifetime). These increases are projected to be particularly pronounced in inland Iberia and Turkey, where static or slightly decreasing frequencies of very high fire danger (figures A2(d) and (e)) would be more than offset by increases in extreme fire danger (figure A3).

Compared with lifetime exposure estimated using the relative, percentile-based indicator, which describes departures from local climatology, the absolute magnitude indicators highlight areas of Southern and Eastern Europe with hotter baseline climates. Estimated increases in lifetime exposure are generally larger when using the percentile-based metric, but increases in exposure based on absolute fire danger thresholds remain substantial in many Southern and Eastern European countries (see tables A4, S4 and S5).

### 3.2. Increasing lifetime exposure to extreme fire weather in Portugal

**Figure 2.**
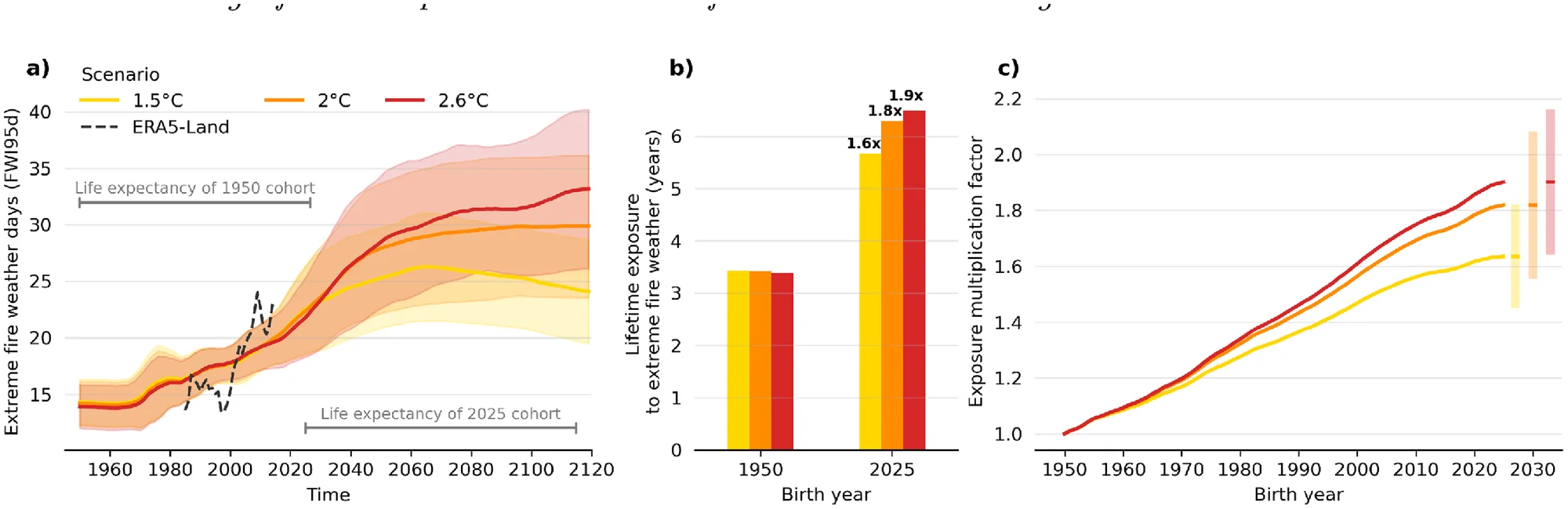
(a) Area-weighted average number of extreme fire weather days (FWI95d) per year in Portugal. Results are shown for three scenarios leading to 1.5$^ {\circ}$ C, 2$^ {\circ}$ C and 2.6$^ {\circ}$ C of warming in 2100, and for ERA5-Land in the 1985–2014 period. Data is smoothed with a 10–year moving average with a minimum window size of 1 year. By definition, 18 extreme fire weather days per year occur in the reference period 1985–2014. (b) Lifetime exposure to extreme fire weather expressed as number of years, for people born in 1950 and 2025 in Portugal. Exposure multiplication factors are indicated above the bars and express the relative lifetime exposure of the 2025 birth year compared to the 1950 birth year. (c) Exposure multiplication factor relative to the lifetime exposure of the 1950 birth year for birth years from 1950–2025 in Portugal, smoothed with a 5-point moving average. In all panels, full lines represent the multi-model mean, and uncertainty spans ± one standard deviation across models.

We analysed projections of extreme fire weather (FWI95d) in Portugal, one of Europe’s most fire-prone countries. We find an increase in the number of extreme fire weather days is projected in Portugal across all assessed warming scenarios (figure 2(a)). These would increase from 14 (12–16, multi-model mean ± 1 SD) days per year in 1950–1970 to 25 (21–29) days under 1.5$^ {\circ}$ C, 30 (24–36) days under 2$^ {\circ}$ C, and 32 (26–38) days per year under a 2.6$^ {\circ}$ C pathway by 2100. Under current policy projections extreme fire weather days in Portugal are thus expected to more than double relative to the 1950–1970 period.

In terms of lifetime exposure, we find people born in 1950, who have a life expectancy of approximately 76.4 years, would experience approximately 3.4 years of their lives under extreme fire weather conditions, with little variation across scenarios (figure 2(b)). Children born in 2025, who have a mean life expectancy of 89.7 years, would instead experience 5.7 years of their life under extreme fire weather conditions in a 1.5$^ {\circ}$ C pathway, 6.3 years in a 2$^ {\circ}$ C pathway and 6.5 years in a 2.6$^ {\circ}$ C pathway (figure 2(b)).

Even under a 1.5$^ {\circ}$ C warming pathway, which implies immediate ambitious global coordinated climate mitigation action [36], people born in 2025 in Portugal are projected to experience 60% more extreme fire weather days than people born in 1950 (figure 2(b)). With global warming reaching 2$^ {\circ}$ C and 2.6$^ {\circ}$ C in 2100, children born in 2025 in Portugal are projected to experience respectively 80% and 90% more extreme fire weather compared to people born in 1950 (figure 2(b)).

We find that climate change is responsible for 74% (64%–84%, multi-model mean ± 1 SD) of the difference in lifetime exposure between people born in 1950 and in 2025 in a 1.5$^ {\circ}$ C scenario, 79% (69%–89%) in a 2$^ {\circ}$ C scenario, and 82% (77%–88%) in a 2.6$^ {\circ}$ C scenario. Life expectancy increases are responsible for the remaining 18%–26% of the difference across warming scenarios. We thus find that climate change is responsible for over three fourths of the difference in exposure between generations across all assessed scenarios, and that this fraction increases with global warming.

Across all warming scenarios, the youngest generations would experience the highest lifetime exposure to fire danger. However, the difference between warming scenarios is also most striking for younger generations (figure 2(c)). This implies that the youngest generations would also have the most to gain from ambitious mitigation. Limiting warming in line with the Paris Agreement target of 1.5$^ {\circ}$ C would reduce the lifetime exposure of Portuguese individuals born in 2025 to extreme fire weather by approximately one year (1.1 (0.5–1.6) years) compared to current policies (figure 2(b)).

**Figure 3.**
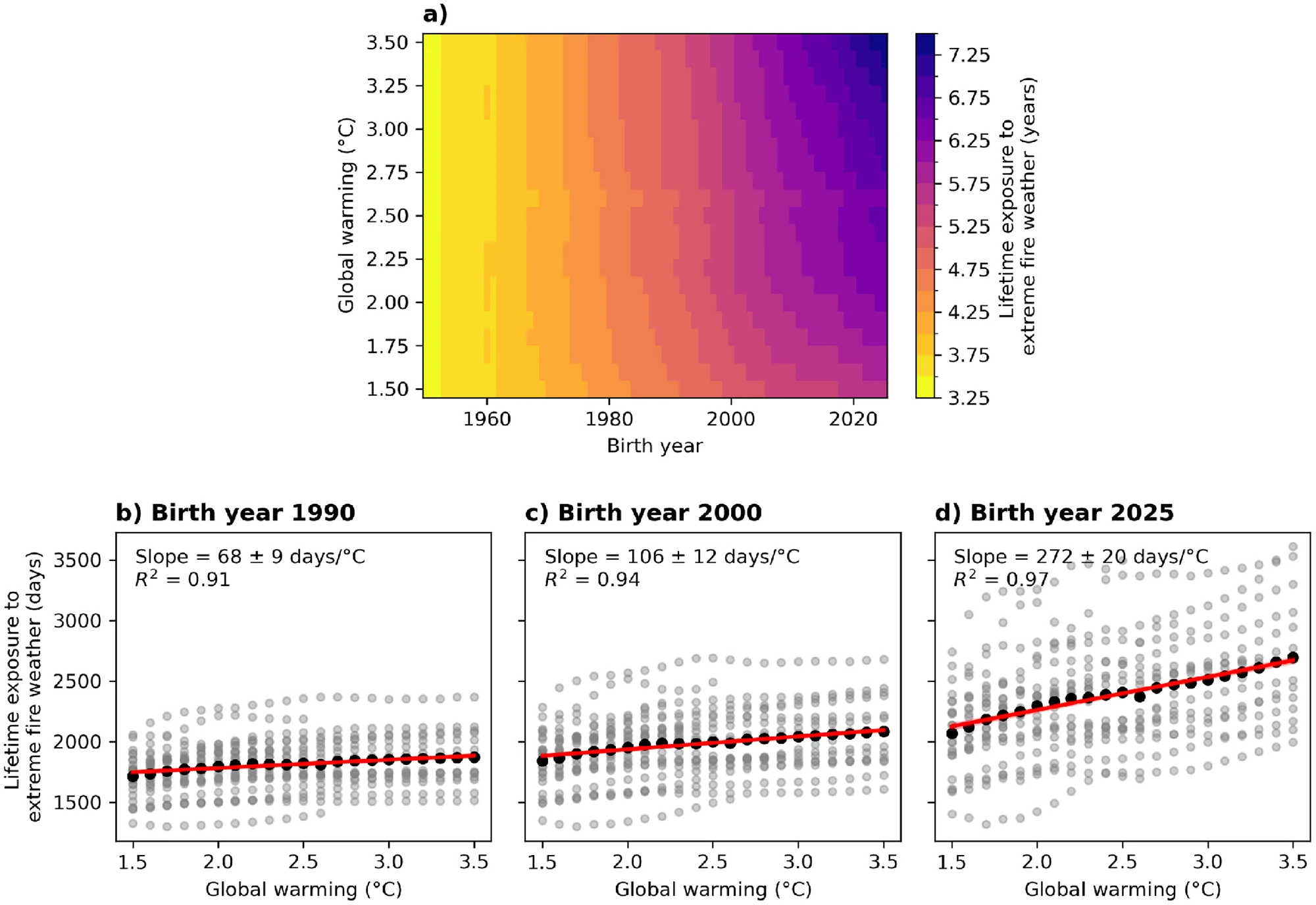
Lifetime exposure to extreme fire weather in Portugal under global warming trajectories reaching 1.5$^ {\circ}$ C–3.5$^ {\circ}$ C in 2100. (a) Years of lifetime exposed for people born in Portugal between 1950 to 2025 under warming pathways from 1.5$^ {\circ}$ C to 3.5$^ {\circ}$ C in 2100. Multi-model mean results are shown. (b)–(d) Additional lifetime exposure per degree of global warming for people born in (b) 1990, (c) 2000, (d) 2025, calculated based on a linear regression of the lifetime exposure against global warming in 2100. Ordinary least squares regression is carried out on the ensemble mean (black dots), individual remapped simulations are shown for context (grey dots). The slope of the linear regression indicates the additional days of lifetime exposure per degree of end-of-century warming (d $^ {\circ}$ C$^ {-1}$). Results for more birth years in Portugal in table A3, and for all countries in table A4. Results for all NUTS3 regions in Portugal in the Supplementary Materials (table S2)), and for all countries, NUTS2 and NUTS3 regions, and birth years, in the Supplementary Data.

Based on our results, we find that, for each birth year, lifetime exposure to extreme fire weather increases approximately linearly with global mean warming in 2100 (figure 3). We can thus estimate the exposure that every additional fraction of a degree of end-of-century warming adds. Based on ordinary-least-squares estimation on the ensemble mean, we find that each degree of warming in 2100 would add approximately 272 d (± 20 d, *R*$^ {2}$ > 0.9) of extreme fire weather to the life of someone born in 2025 in Portugal, 106 d (± 12, ${{\textit{R}}}^{2} > 0.9$) for someone born 2000 and 68 d (± 9, *R*$^ {2}$ > 0.9) for someone born in 1990 (figures 3(b)–(d)), see results for all countries in table A4. As in previous results, the sensitivity to higher warming is highest for the youngest generations.

When considering absolute thresholds from the EFFIS fire danger forecast [60] (figures A2 and A3), we find that, in Portugal, each additional degree of warming by 2100 would add 248 d (± 16 d, *R*$^2 > 0.9$) of very high fire danger (38 $\unicode{x2A7D}$ FWI < 50) to the lifetime exposure of someone born in 2025. For extreme fire danger (FWI $\unicode{x2A7E}$ 50), the corresponding increase is 88 d (± 7 d, *R*$^2 > 0.9$). Results for additional birth years are shown in tables S2 and S3, and results for all European countries in tables S4 and S5.

On average across Portugal, the sensitivity of lifetime exposure to very high fire danger (38 $\unicode{x2A7D}$ FWI < 50) to end-of-century warming is broadly comparable to that found for extreme fire weather defined using the 95th percentile threshold (FWI95d). By contrast, the sensitivity is lower for extreme fire danger (FWI $\unicode{x2A7E}$ 50). Spatially, lifetime exposure to fire danger based on absolute thresholds is highest in Southern Portugal, where the baseline climate is hotter and drier.

### 3.3. Sub-national variation in increasing lifetime exposure to extreme fire weather in Portugal

**Figure 4.**
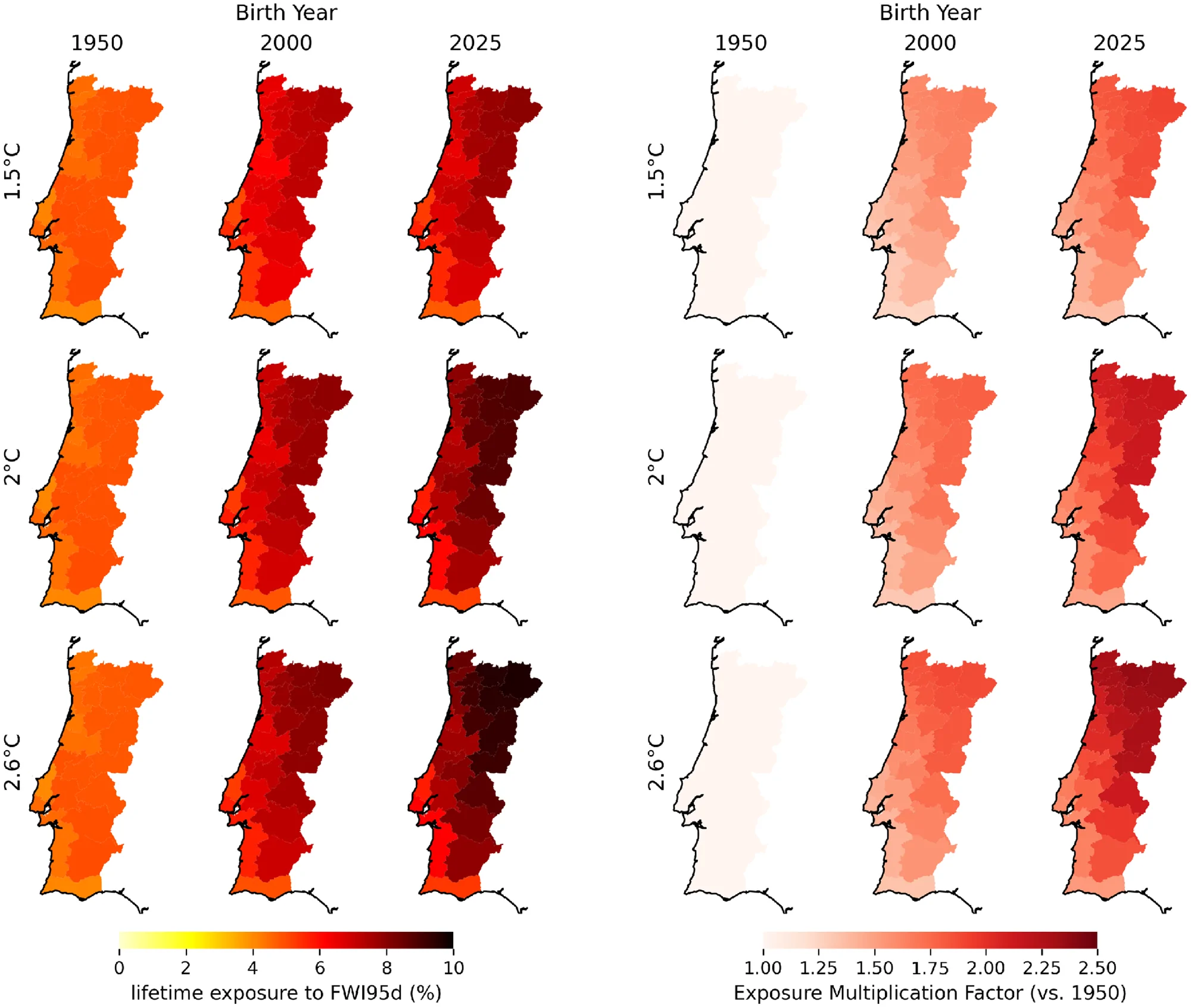
Percentage of lifetime exposed to extreme fire weather (FWI95d, left). Exposure multiplication factor relative to the lifetime exposure of the 1950 birth year (right). Results are shown at the NUTS3 level of Portugal for people born in 1950, 2000 and 2025, under 1.5$^ {\circ}$ C, 2$^ {\circ}$ C and 2.6$^ {\circ}$ C warming pathways. Multi-model mean is shown.

Analysing projections at the NUTS3 level in Portugal, we find that lifetime exposure to extreme fire weather (FWI95d) is projected to increase across the whole country, with the greatest increases in the Northeast (Norte and Centro regions), especially inland where oceanic influence is limited (figure 4, left). While people born in 1950 would 4%–5% (multi-model mean, range across regions) of their lifetime in extreme fire weather conditions, with little variation across scenarios, we find people born in 2025 are projected to live 5%–8% of their lifetime exposed across regions in a 1.5$^ {\circ}$ C scenario, 5%–9% in a 2$^ {\circ}$ C scenario and 5%–10% under current policies (figure 4, left).

Across regions, we find people born in 2025 are projected to experience 40%–90% more extreme fire weather during their lifetime than people born in 1950 in a 1.5$^ {\circ}$ C pathway (figure 4, right), 50% more to 2.1 times as much in a 2$^ {\circ}$ C scenario and 50% more to 2.4 times as much in a current policies pathway, with the highest increases in the Northeast (figure 4, right).

**Figure 5.**
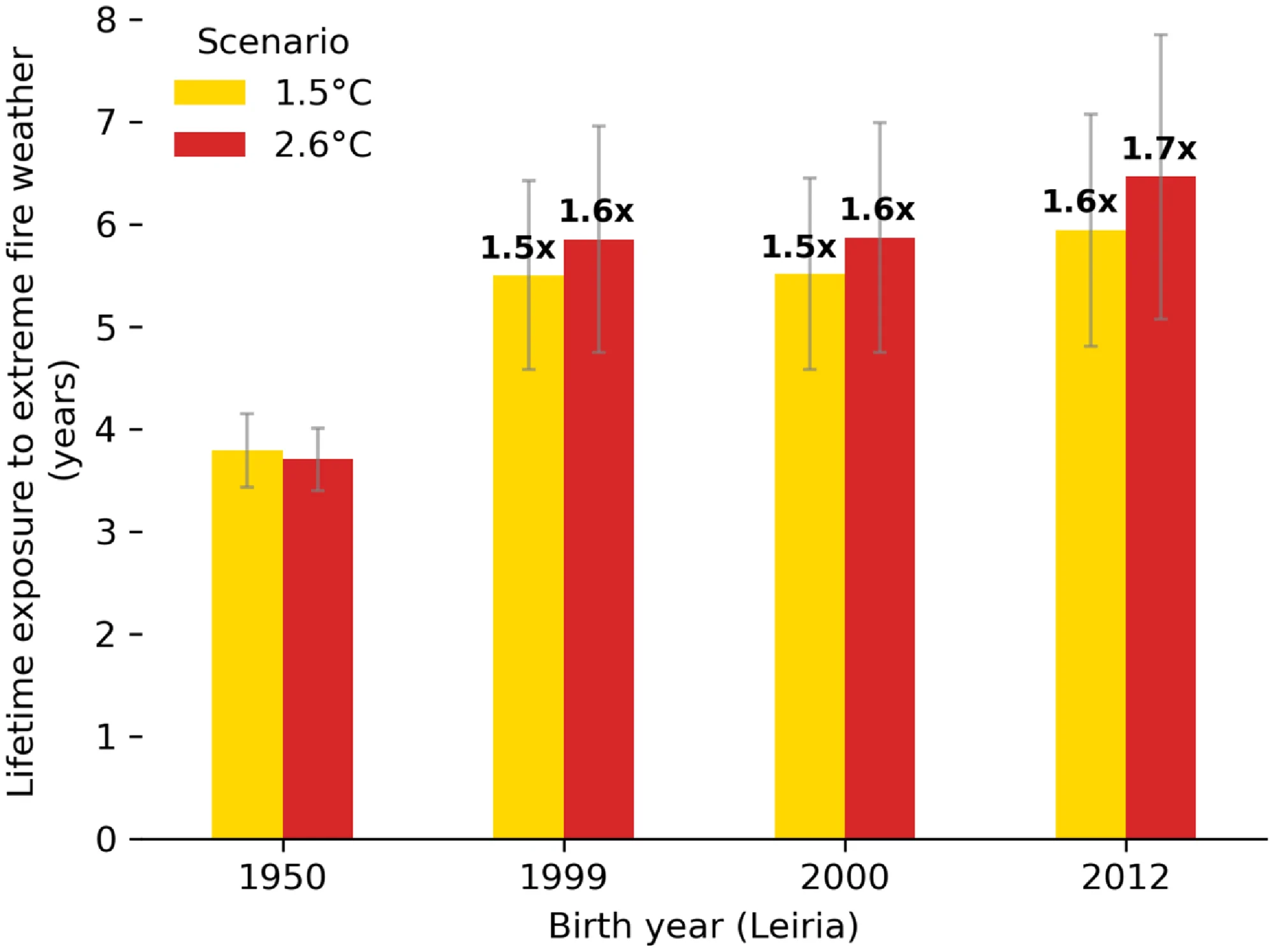
Lifetime exposure to extreme fire weather (FWI95d) in the NUTS3 region of Leiria, Portugal. Results are shown for people born in 1950, 1999, 2000 and 2012, under 1.5$^ {\circ}$ C and 2.6$^ {\circ}$ C warming pathways. Multi-model mean plus and minus one standard deviation is shown. Multiplication factors express the multi-model mean relative exposure of each birth year compared to the 1950 birth year under the same pathway.

We focus on one region as a case study, Leiria, located in the coastal part of the Centro region, as this region was heavily affected in recent fire seasons, notably in 2017 (figures S8(c) and (d)). Here, we find people born in 1950 are projected to live approximately 3.8 years of their life in extreme fire weather conditions (figure 5). People born in 1999 are projected to live 50% more time in these conditions (5.5 years) in a 1.5$^ {\circ}$ C pathway and 60% more (5.9 years) in a current policies pathway (figure 5). Children born in 2012 are projected to live respectively 60% more (5.9 years) and 70% more time (6.5 years) in extreme fire weather conditions than people born in 1950 in 1.5$^ {\circ}$ C and 2.6$^ {\circ}$ C pathways (figure 5).

## 4. Discussion and conclusions

Our results show that, irrespective of the warming pathway, climate change is increasing lifetime exposure to extreme fire weather for young generations across Europe. However, both the magnitude of the increase and the resulting intergenerational disparity in exposure are greater under higher warming pathways. Under a current policies scenario, projected to lead to 2.6$^ {\circ}$ C of warming by 2100 [58], the youngest generations in Europe are projected to experience on average 3 additional years of exposure compared to their older counterparts. Limiting warming to 1.5$^ {\circ}$ C would reduce the exposure of younger generations, for instance by cutting the exposure of people born in 2025 in Portugal by over a year relative to current policies. Although some degree of intergenerational inequity is thus committed due to past emissions and plausible future pathways, there remains scope to reduce future inequities through ambitious mitigation.

Our findings have implications for intergenerational justice, as we show that younger generations, who have contributed least to historical emissions, are projected to bear a disproportionate share of future exposure to extreme fire weather. Such analyses can inform the arguments of youth climate justice movements and legal initiatives asking for more ambitious climate action [32, 61].

We find the largest increases in exposure projected for young people in Southern Europe, a region that is already highly fire-prone (figure 1) [19, 62]. This is in line with previous research [2, 24, 62], and has been linked to projections of hotter and drier conditions in the Mediterranean [26–28, 62] and longer fire seasons [63]. Together with the high impacts of recent fire events in Southern Europe [6, 8, 14], our findings raise concerns for the region and underline the growing importance of effective land and fire management and preventative measures in fire-prone areas, together with climate mitigation.

Portugal has emerged as one of the most fire-prone countries in Europe [6, 18–20]. We find a pronounced projected increase in lifetime exposure to extreme fire weather for young people in Portugal, with the largest increases in the Northeast (figure 4), which is already today one of the most fire-prone regions in Europe [6, 19, 62, 64]. While the Southern Alentejo region experiences the highest absolute fire weather values due to a drier and hotter climate (figure S1), wildfires mostly affect the forested Central and Northern regions S8(a) and (b). Historical patterns of high fire risk and projected increases in fire weather raise concerns on heightened fire danger for communities and ecosystems in these areas. Our findings are in line with previous research that has projected high increases in fire weather [63, 65] and activity [62, 66] in the Northwest of the Iberian Peninsula, associated with projections of strong increases in summer maximum temperatures [28, 30, 65, 67] and possible declines in summer precipitation [65, 68].

Using absolute magnitude indicators of fire danger classes, we find very high and extreme fire danger is projected to be concentrated in Southern Europe, where baseline climates are hotter and drier. Increases in lifetime exposure to these classes are expected to be highest in Southern Europe, but occur also in other parts of the continent under higher warming pathways (figures A2 and A3).

With *dem4cli* we present a new community Python package that can be used to estimate exposure, including age-specific and lifetime exposure to any climate hazard. This package expands on previous research [34, 35], increasing the robustness in the emulation approach and flexibility for user-defined analyses. The lifetime exposure method allows to compare the projected exposure to climate hazards of people in different locations and born in different years, at national and sub-national scales, along policy-relevant warming pathways.

Several aspects of this study could be improved through further research. First, we study fire weather, namely the hot-dry-windy meteorological conditions that are conducive to the initiation and spread of fires, not actual fire occurrence. Other factors such as vegetation, land cover and management, and human factors are important in determining the occurrence and spread of fires [21, 41, 69], and mediate the local sensitivity of fires to climatic conditions [23, 70]. Nonetheless, the FWI has been shown to be a good proxy of fire danger in many locations [3, 23] including Europe [19, 39, 62, 71], the Mediterranean [39, 72] and Portugal [41]. Moreover, we do not consider vegetation–climate feedbacks that could alter pyroregion structure and fire-weather relationships [66, 70]. However, given uncertainties in process-based fire models [3, 73, 74] and future projections of biogeography, land use, and management [75], projections of meteorological fire danger remain valuable.

Second, we use an ensemble of bias-adjusted and statistically downscaled simulations from the latest state-of-the-art CMIP6 GCMs, from [24] (table A1). For each stylized warming pathway, we pool simulations across SSP experiments (table A2), meaning the spread in results in each warming pathway includes both model uncertainty and uncertainty from natural variability. By aggregating fire-weather exposure over the multi-decadal lifespans of individuals, our analysis focuses primarily on the forced response of change, reducing the relative contribution of natural variability compared with analyses at shorter time scales [76]. Nonetheless, future studies could use larger ensembles of GCMs to better represent inter-model differences in regional fire-weather responses to specific global warming levels, as well as multiple initial-condition ensemble members to more fully capture the possible range of uncertainty arising from natural variability. Future studies could also use dynamically downscaled simulations from the Coordinated Regional Climate Downscaling Experiment, including the latest generation of simulations that are currently becoming available [65]. Moreover, the previous generation of GCMs (CMIP5) underestimated FWI trends in the Mediterranean [3], while global and regional climate models underestimate warming over Europe in the historical period [27, 77–79], meaning our results could be conservative.

Finally, we use an emulation approach to model the regional response to policy-relevant global warming pathways. By using a GMT-mapping approach, we assume that the GMT-to-hazard relationship is path-independent and that the hazard magnitude and regional response depends mainly on GMT. This is supported for various indicators [45, 49, 80, 81] and similar reasoning is applied in other emulation approaches [50, 82–85], but we note that this might be less applicable in overshoot or temperature stabilization scenarios [86–90], or where climate is strongly affected by aerosol concentrations or land use changes [91, 92]. Future work could specify different relationships before and after peak warming in the context of overshoot, and could increase policy relevance by emulating exposure under emission pathways, instead of warming pathways.

In conclusion, our results show that exposure to extreme fire weather accumulates over decades, creating substantial risk for today’s young generations across their lifetimes. Policy decisions on emission reductions taken today will shape the exposure to hazardous conditions of children and of future generations. By explicitly considering lifetime exposure in risk assessments, this study helps clarify the long-term consequences of climate change and highlights the benefits of prevention compared with the costs of delayed action. Our results show that every fraction of warming avoided reduces the burden on young generations across Europe by lowering their lifetime exposure to extreme and dangerous fire weather. Climate change mitigation should therefore be understood as a preventative policy to protect the health and safety of today’s young generations.

## Data Availability

Dem4cli is publicly available here: https://github.com/rosapietroiusti/dem4cli/ [93]. All code necessary to reproduce the analyses and figures is available here: https://github.com/rosapietroiusti/fwi-portugal. All data to reproduce the analyses is publicly available. Burned area data are available via the European Forest Fire Information System–EFFIS (https://forest-fire.emergency.copernicus.eu) of the European Commission Joint Research Centre. Life expectancy data and cohort size data are available from the UN Wold Population Prospects 2024 (https://population.un.org/wpp/). Gridded population data are publicly available here: https://doi.org/10.5281/zenodo.17572467. Country shapefiles are publicly available via Natural Earth. European NUTS-level shapefiles are publicly available via Eurostat (https://ec.europa.eu/eurostat/web/gisco/geodata/statistical-units/territorial-units-statistics). The data that support the findings of this study are openly available at the following URL/DOI:. https://zenodo.org/records/12095573. Supplementary data includes lifetime exposure and additional days per degree of warming, for all fire weather metrics used in paper, for all European countries, NUTS2 and NUTS3 regions. Supplementary data 1 available https://doi.org/10.1088/1748-9326/ae93de/data1. Supplementary data 2 available https://doi.org/10.1088/1748-9326/ae93de/data2. Supplementary data 3 available https://doi.org/10.1088/1748-9326/ae93de/data3. Supplementary data 4 available https://doi.org/10.1088/1748-9326/ae93de/data4. Supplementary data 5 available https://doi.org/10.1088/1748-9326/ae93de/data5. Supplementary data 6 available https://doi.org/10.1088/1748-9326/ae93de/data6. Supplementary data 7 available https://doi.org/10.1088/1748-9326/ae93de/data7. Supplementary data 8 available https://doi.org/10.1088/1748-9326/ae93de/data8. Supplementary data 9 available https://doi.org/10.1088/1748-9326/ae93de/data9. Supplementary data 10 available https://doi.org/10.1088/1748-9326/ae93de/data10.

## Appendix

### A.1. Appendix tables

**Table A1.** List of GCMs, ensemble members and experiments used in this study. All data was bias adjusted and statistically downscaled against ERA5-Land prior to calculation of the FWI, as described in [24].

GCM	Ensemble member	Experiments
ACCESS-CM2	r1i1p1f1	historical, SSP126, SSP245, SSP370, SSP585
CNRM-ESM2-1	r1i1p1f2	historical, SSP126, SSP245, SSP370, SSP585
CanESM5	r1i1p1f1	historical, SSP126, SSP245, SSP370, SSP585
EC-Earth3	r1i1p1f1	historical, SSP126, SSP245, SSP370, SSP585
MPI-ESM1-2-HR	r1i1p1f1	historical, SSP126, SSP245, SSP370, SSP585
MRI-ESM2-0	r1i1p1f1	historical, SSP126, SSP245, SSP370, SSP585

**Table A2.** Number of individual simulations contributing to each stylized warming pathway by model and total. Columns indicate stylized warming pathways by their GMT anomaly in 2100, rows indicate the number of individual simulations for each model (from SSP126, SSP245, SSP370, SSP585) that inform each stylized warming pathway, and the total across all models. The ensemble size is smaller for higher-warming pathways, due to lower-warming simulations not reaching high GMT levels.

Stylized warming pathway ($^\circ$ C)	1.5	1.6	1.7	1.8	1.9	2.0	2.1	2.2	2.3	2.4	2.5	2.6	2.7	2.8	2.9	3.0	3.1	3.2	3.3	3.4	3.5
ACCESS-CM2	4	4	4	4	4	4	4	4	4	4	4	4	4	4	3	3	3	3	3	3	3
CNRM-ESM2-1	4	4	4	4	4	4	4	4	4	3	3	3	3	3	3	3	3	3	2	2	2
CanESM5	4	4	4	4	4	4	4	4	4	4	4	4	3	3	3	3	3	3	3	3	3
EC-EARTH	4	4	4	4	4	4	4	4	4	3	3	3	3	3	3	3	3	3	2	2	2
MPI-ESM1-2-HR	4	4	4	4	3	3	3	3	3	3	3	2	2	2	2	2	2	2	2	2	2
MRI-ESM2-0	4	4	4	4	4	4	4	4	3	3	3	3	3	3	2	2	2	2	2	2	2
Total	24	24	24	24	23	23	23	23	22	20	20	19	18	18	16	16	16	16	14	14	14

**Table A3.** Additional days of extreme fire weather (FWI95d) experienced per degree of end-of-century warming (d $^ {\circ}$ C$^ {-1}$), in Portugal, for selected birth years. Best estimate ± 95% confidence interval. For results for all birth years see supplementary data Excel files.

Birth year	d$^ {\circ}$ C$^ {-1}$	*R* $^2$
1990	68 (± 9)	0.91
1999	103 (± 12)	0.94
2000	106 (± 12)	0.94
2010	162 (± 15)	0.96
2012	174 (± 15)	0.96
2020	231 (± 18)	0.97
2025	272 (± 20)	0.97

**Table A4.** Additional days of extreme fire weather (FWI95d) experienced per degree of end-of-century warming (d$^ {\circ}$ C$^ {-1}$), for selected birth years and all countries. Best estimate ± 95% confidence interval. All ${{\textit{R}}}^2 {\unicode{x2A7E}}0.79$. For results for all birth years, NUTS2 and NUTS3 regions see Supplementary Data files.

Country	1990	2000	2010	2025
Albania	119 (± 10)	196 (± 12)	290 (± 14)	477 (± 18)
Austria	82 (± 12)	135 (± 16)	199 (± 19)	317 (± 24)
Belgium	126 (± 16)	206 (± 20)	289 (± 24)	432 (± 34)
Bulgaria	117 (± 10)	200 (± 10)	309 (± 10)	514 (± 12)
Croatia	85 (± 9)	156 (± 11)	245 (± 13)	410 (± 18)
Cyprus	26 (± 4)	45 (± 5)	68 (± 5)	107 (± 6)
Czechia	68 (± 11)	112 (± 17)	167 (± 22)	270 (± 28)
Denmark	79 (± 9)	129 (± 12)	183 (± 14)	264 (± 17)
Estonia	23 (± 5)	58 (± 10)	106 (± 13)	177 (± 15)
Finland	41 (± 5)	71 (± 8)	107 (± 9)	161 (± 10)
France	117 (± 13)	192 (± 18)	277 (± 22)	431 (± 28)
Germany	95 (± 11)	158 (± 15)	222 (± 18)	338 (± 24)
Greece	125 (± 5)	204 (± 5)	297 (± 6)	473 (± 8)
Hungary	78 (± 11)	130 (± 17)	202 ( ± 21)	336 ( ± 25)
Iceland	108 ( ± 8)	154 ( ± 10)	198 ( ± 13)	263 ( ± 18)
Ireland	72 ( ± 13)	111 ( ± 18)	153 ( ± 23)	220 ( ± 29)
Italy	108 ( ± 9)	187 ( ± 10)	279 ( ± 12)	448 ( ± 15)
Latvia	29 ( ± 4)	66 ( ± 7)	117 ( ± 11)	191 ( ± 13)
Liechtenstein	85 ( ± 9)	142 ( ± 13)	207 ( ± 16)	321 ( ± 19)
Lithuania	53 ( ± 6)	91 ( ± 9)	145 ( ± 13)	223 ( ± 17)
Luxembourg	116 ( ± 16)	200 ( ± 21)	297 ( ± 27)	457 ( ± 34)
Malta	14 ( ± 1)	24 ( ± 2)	38 ( ± 3)	58 ( ± 4)
Montenegro	118 ( ± 10)	188 ( ± 12)	290 ( ± 15)	479 ( ± 20)
Netherlands	111 ( ± 12)	176 ( ± 15)	243 ( ± 18)	353 ( ± 26)
North Macedonia	133 ( ± 10)	216 ( ± 11)	327 ( ± 14)	535 ( ± 21)
Norway	57 ( ± 3)	91 ( ± 5)	127 ( ± 6)	179 ( ± 8)
Poland	66 ( ± 10)	112 ( ± 15)	168 ( ± 20)	258 ( ± 24)
Portugal	68 ( ± 9)	106 ( ± 12)	163 ( ± 15)	272 ( ± 20)
Romania	90 ( ± 13)	167 ( ± 16)	271 ( ± 19)	449 ( ± 19)
Serbia	100 ( ± 9)	168 ( ± 9)	275 ( ± 11)	466 ( ± 14)
Slovakia	85 ( ± 17)	131 ( ± 22)	197 ( ± 27)	321 ( ± 33)
Slovenia	91 ( ± 9)	159 ( ± 12)	252 ( ± 16)	417 ( ± 23)
Spain	113 ( ± 9)	185 ( ± 11)	281 ( ± 13)	448 ( ± 14)
Sweden	62 ( ± 8)	107 ( ± 11)	156 ( ± 12)	236 ( ± 13)
Switzerland	97 ( ± 11)	161 ( ± 16)	237 ( ± 20)	376 ( ± 25)
Türkiye	103 ( ± 6)	176 ( ± 7)	270 ( ± 7)	435 ( ± 8)
United Kingdom	98 ( ± 16)	155 ( ± 21)	214 ( ± 26)	312 ( ± 33)

Countries marked with a ^†^ are only partially covered by our data (see supplementary figure S4 for coverage).

## References

[1] Turco M, Moreno A, Garnés-Morales G, Torres-Vázquez M A, Di Giuseppe F, Bedia J, Herrera S, Provenzale A, Abatzoglou J T (2026). The emerging human fingerprint on global extreme fire weather. Sci. Adv..

[2] Abatzoglou J T, Williams A P, Barbero R (2019). Global Emergence of anthropogenic climate change in fire weather indices. Geophys. Res. Lett..

[3] Jones M W (2022). Global and regional trends and drivers of fire under climate change. Rev. Geophys..

[4] Jain P, Castellanos-Acuna D, Coogan S C P, Abatzoglou J T, Flannigan M D (2022). Observed increases in extreme fire weather driven by atmospheric humidity and temperature. Nat. Clim. Change.

[5] Abatzoglou J T, Williams A P (2016). Impact of anthropogenic climate change on wildfire across western US forests. Proc. Natl Acad. Sci..

[6] Sánchezá-Hernández G (2025). Record-Breaking 2025 European wildfires concentrated in Northwest Iberia. Glob. Change Biol..

[7] Keeping T (2025). Extreme fire weather conditions in Spain and Portugal now common due to climate change.

[8] Keeping T (2025). Weather conditions leading to deadly wildfires in Türkiye, Cyprus and Greece made 10 times more likely due to climate change.

[9] JRC (2022). Forest fires in Europe, Middle East and North Africa 2021. European Commission.

[10] JRC (2023). Forest fires in Europe, Middle East and North Africa 2022. European Commission.

[11] JRC (2024). Forest fires in Europe, Middle East and north Africa 2023. European Commission.

[12] JRC (2025). Advance report on forest fires in Europe, middle east and North Africa 2024. European Commission.

[13] Jones M W (2024). State of wildfires 2023–2024. Earth Syst. Sci. Data.

[14] Turco M, Jerez S, Augusto S, Tarín-Carrasco P, Ratola N, Jiménez-Guerrero P, Trigo R M (2019). Climate drivers of the 2017 devastating fires in Portugal. Sci. Rep..

[15] Ramos A M, Russo A, DaCamara C C, Nunes S, Sousa P, Soares P, Lima M M, Hurduc A, Trigo R M (2023). The compound event that triggered the destructive fires of October 2017 in Portugal. iScience.

[16] Grünig M, Seidl R, Senf C (2023). Increasing aridity causes larger and more severe forest fires across Europe. Glob. Change Biol..

[17] JRC (2020). European Wildfire Danger and Vulnerability in a Changing Climate: Towards Integrating Risk Dimensions : JRC Peseta IV Project: Task 9 Forest Fires.

[18] EFFIS (2026). EFFIS estimates for European Union. https://forest-fire.emergency.copernicus.eu/apps/effis.statistics/estimates.

[19] Galizia L F, Curt T, Barbero R, Rodrigues M (2022). Understanding fire regimes in Europe. Int. J. Wildland Fire.

[20] Kelley D I (2025). State of wildfires 2024–2025. Earth Syst. Sci. Data.

[21] Pausas J G, Fernández-Muñoz S (2012). Fire regime changes in the western Mediterranean basin: from fuel-limited to drought-driven fire regime. Clim. Change.

[22] Turco M, Hardenberg J V, AghaKouchak A, Llasat M C, Provenzale A, Trigo R M (2017). On the key role of droughts in the dynamics of summer fires in Mediterranean Europe. Sci. Rep..

[23] Abatzoglou J T, Williams A P, Boschetti L, Zubkova M, Kolden C A (2018). Global patterns of interannual climate–fire relationships. Glob. Change Biol..

[24] Hetzer J, Forrest M, Ribalaygua J, Prado-López C, Hickler T (2024). The fire weather in Europe: large-scale trends towards higher danger. Environ. Res. Lett..

[25] Garroussi S E, Di Giuseppe F, Barnard C, Wetterhall F (2024). Europe faces up to tenfold increase in extreme fires in a warming climate. npj Clim. Atmos. Sci..

[26] Cos J, Doblas-Reyes F, Jury M, Marcos R, Bretonnière P-A, Samsó M (2022). The Mediterranean climate change hotspot in the CMIP5 and CMIP6 projections. Earth Syst. Dyn..

[27] IPCC, IPCC (2021). Chapter 10: Linking global to regional climate change. Climate Change 2021–The Physical Science Basis: Working Group I Contribution to the Sixth Assessment Report of the Intergovernmental Panel on Climate Change.

[28] Coppola E (2021). Assessment of the European Climate Projections as Simulated by the Large EURO–CORDEX regional and global climate model ensemble. J. Geophys. Res.: Atmos..

[29] Turco M, Rosa-Cánovas J J, Bedia J, Jerez S, Montávez J P, Llasat M C, Provenzale A (2018). Exacerbated fires in Mediterranean Europe due to anthropogenic warming projected with non-stationary climate-fire models. Nat. Commun..

[30] Lionello P, Scarascia L (2018). The relation between climate change in the Mediterranean region and global warming. Reg. Environ. Change.

[31] Cramer W (2018). Climate change and interconnected risks to sustainable development in the Mediterranean. Nat. Clim. Change.

[32] Rogelj J, Willers M (2021). Youth activists are forcing governments to take account of the intergenerational impact of climate change. CCLS Energy and Climate Change Law Institute Review.

[33] UN Committee on the Rights of the Child (2023). ‘General Comment No 26 (2023) on Children’s Rights and the Environment, with a Special Focus on Climate Change’ UN Doc CRC/C/GC/26.

[34] Thiery W (2021). Intergenerational inequities in exposure to climate extremes. Science.

[35] Grant L, Vanderkelen I, Gudmundsson L, Fischer E, Seneviratne S I, Thiery W (2025). Global emergence of unprecedented lifetime exposure to climate extremes. Nature.

[36] IPCC, IPCC (2022). Chapter 3: Mitigation Pathways Compatible with Long-term Goals. Climate Change 2022 - Mitigation of Climate Change.

[37] Low S, Brutschin E, Baum C M, Sovacool B K (2025). Expert perspectives on incorporating justice considerations into integrated assessment modelling. npj Clim. Action.

[38] Van Wagner C E 1987 Development and structure of the canadian forest fire weather index system. Ser. forestry technical report (Minister of Supply and Services Canada).

[39] Di Giuseppe F, Vitolo C, Krzeminski B, Barnard C, Maciel P, San-Miguel J (2020). Fire weather index: the skill provided by the European Centre for Medium-Range Weather Forecasts ensemble prediction system. Nat. Hazards Earth Syst. Sci..

[40] Vitolo C, Di Giuseppe F, Barnard C, Coughlan R, San-Miguel-Ayanz J, Libertá G, Krzeminski B (2020). ERA5-based global meteorological wildfire danger maps. Sci. Data.

[41] Carvalho A, Flannigan M D, Logan K, Miranda A I, Borrego C (2008). Fire activity in Portugal and its relationship to weather and the Canadian Fire Weather Index System. Int. J. Wildland Fire.

[42] Quilcaille Y, Batibeniz F, Ribeiro A F S, Padrón R S, Seneviratne S I (2023). Fire weather index data under historical and shared socioeconomic pathway projections in the 6th phase of the Coupled Model Intercomparison Project from 1850 to 2100. Earth Syst. Sci. Data.

[43] CEMS (2019). Fire danger indices historical data from the Copernicus emergency management service.

[44] Pietroiusti R, Fischer E M, Stuart-Smith R F, Harrington L J, Brimicombe C, Mengel M, Savaresi A, Adelman S, Thiery W (2026). Age-specific exposure to human-induced increases in humid heat. Sci. Adv..

[45] Hausfather Z, Marvel K, Schmidt G A, Nielsen-Gammon J W, Zelinka M (2022). Climate simulations: recognize the ‘hot model’ problem. Nature.

[46] Byers E (2022). AR6 scenarios database.

[47] Kikstra J S (2022). The IPCC sixth assessment report WGIII climate assessment of mitigation pathways: from emissions to global temperatures. Geosci. Model Dev..

[48] Schleussner C-F, Ganti G, Rogelj J, Gidden M J (2022). An emission pathway classification reflecting the Paris Agreement climate objectives. Commun. Earth Environ..

[49] Herger N, Sanderson B M, Knutti R (2015). Improved pattern scaling approaches for the use in climate impact studies. Geophys. Res. Lett..

[50] Quilcaille Y, Gudmundsson L, Seneviratne S I (2023). Extending MESMER-X: a spatially resolved Earth System Model emulator for fire weather and soil moisture. Earth Syst. Dyn..

[51] Paprotny D (2025). Exposure datasets at multiple scales. Horizon Europe project COMPASS. Deliverable D3.1. https://compass-climate.eu/Public%20Deliverables/D3.1_Exposure%20datasets%20at%20multiple%20scales.pdf.

[52] Van Vuuren D (2025). The Scenario model intercomparison project for CMIP7 (ScenarioMIP-CMIP7). Geosci. Model Dev..

[53] KC S (2024). Updating the Shared Socioeconomic Pathways (SSPs) Global Population and Human Capital Projections. IIASA Working Paper.

[54] KC S (2025). Population and human capital projections by IIASA and Wittgenstein Center (WIC) v3.2 (May 2025).

[55] UNDESA (2024). World population prospects 2024, United Nations, Department of Economic and Social Affairs, Population Division. https://population.un.org/wpp/.

[56] Goldstein J R, Wachter K W (2006). Relationships between period and cohort life expectancy: Gaps and lags. Popul. Stud..

[57] Eurostat (2018). Nomenclature of Territorial Units for Statistics (NUTS) 2016 – Statistical Units – Data Set (GISCO). https://gisco-services.ec.europa.eu/distribution/v2/nuts/.

[58] UNEP (2025). Emissions Gap Report 2025: Off Target - Continued Collective inaction puts Global Temperature Goal at Risk. United Nations Environment Programme. Tech. Rep..

[59] C A T (2025). Climate action tracker: warming projections global update.

[60] EFFIS (2025). Fire danger forecast. https://forest-fire.emergency.copernicus.eu/about-effis/technical-background/fire-danger-forecast.

[61] Parker L, Mestre J, Wewerinke-Singh M (2021). When the kids put climate change on trial youth-focused rights-based climate litigation around the world. J. Hum. Rights Environ..

[62] Galizia L F, Barbero R, Rodrigues M, Ruffault J, Pimont F, Curt T (2023). Global warming reshapes European pyroregions. Earth’s Future.

[63] Bento V A, Lima D C, Santos L C, Lima M M, Russo A, Nunes S A, DaCamara C C, Trigo R M, Soares P M (2023). The future of extreme meteorological fire danger under climate change scenarios for Iberia. Weather Clim. Extrem..

[64] Trigo R M, Sousa P M, Pereira M G, Rasilla D, Gouveia C M (2016). Modelling wildfire activity in Iberia with different atmospheric circulation weather types. Int. J. Climatol..

[65] Pereira S C, Monteiro N, Vaz R, Carvalho D (2024). High-resolution projections of future FWI conditions for Portugal according to CMIP6 future climate scenarios. Theor. Appl. Climatol..

[66] Calheiros T, Pereira M, Nunes J (2021). Assessing impacts of future climate change on extreme fire weather and pyro-regions in Iberian Peninsula. Sci. Total Environ..

[67] Cardoso R M, Soares P M M, Lima D C A, Miranda P M A (2019). Mean and extreme temperatures in a warming climate: EURO CORDEX and WRF regional climate high-resolution projections for Portugal. Clim. Dyn..

[68] Soares P M M, Cardoso R M, Lima D C A, Miranda P M A (2017). Future precipitation in Portugal: high-resolution projections using WRF model and EURO-CORDEX multi-model ensembles. Clim. Dyn..

[69] Oliveira S, Gonçalves A, Zêzere J L (2021). Reassessing wildfire susceptibility and hazard for mainland Portugal. Sci. Total Environ..

[70] Pausas J G, Paula S (2012). Fuel shapes the fire—climate relationship: evidence from Mediterranean ecosystems. Glob. Ecol. Biogeogr..

[71] Forrest M (2024). Understanding and simulating cropland and non-cropland burning in Europe using the BASE (burnt area simulator for europe) model. Biogeosciences.

[72] Viegas D X, Bovio G, Ferreira A, Nosenzo A, Sol B (1999). Comparative study of various methods of fire danger evaluation in southern Europe. Int. J. Wildland Fire.

[73] Teckentrup L (2019). Response of simulated burned area to historical changes in environmental and anthropogenic factors: a comparison of seven fire models. Biogeosciences.

[74] Burton C (2024). Global burned area increasingly explained by climate change. Nat. Clim. Change.

[75] De Hertog S J (2024). Effects of idealized land cover and land management changes on the atmospheric water cycle. Earth Syst. Dyn..

[76] Hawkins E, Sutton R (2009). The potential to narrow uncertainty in regional climate predictions. Bull. Am. Meteorol. Soc..

[77] Vautard R (2023). Heat extremes in western Europe increasing faster than simulated due to missed atmospheric circulation changes. Nat. Commun..

[78] Schumacher D L, Singh J, Hauser M, Fischer E M, Wild M, Seneviratne S I (2024). Exacerbated summer European warming not captured by climate models neglecting long-term aerosol changes. Commun. Earth Environ..

[79] Singh J, Sippel S, Fischer E M (2023). Circulation dampened heat extremes intensification over the Midwest USA and amplified over Western Europe. Commun. Earth Environ..

[80] Schleussner C-F (2016). Differential climate impacts for policy-relevant limits to global warming: the case of 1.5$^\circ$C and 2$^\circ$C. Earth Syst. Dyn..

[81] Tebaldi C, Arblaster J M (2014). Pattern scaling: its strengths and limitations, and an update on the latest model simulations. Clim. Change.

[82] Byers E, Werning M, Perrette M, Schwind N, Krey V, Riahi K, Schleussner C-F (2025). Fast climate impact emulation for global temperature scenarios with the rapid impact model emulator (RIME). Env. Res: Clim..

[83] Schwind N (2025). RIME-X v1.0: combining simple climate models, earth system models, and climate impact models into a unified statistical emulator for regional climate indicators. Geosci. Model Dev..

[84] Tebaldi C, Snyder A, Dorheim K (2022). STITCHES: creating new scenarios of climate model output by stitching together pieces of existing simulations. Earth Syst. Dyn..

[85] Schwaab J (2024). Spatially resolved emulated annual temperature projections for overshoot pathways. Sci. Data.

[86] Pfleiderer P, Schleussner C-F, Sillmann J (2024). Limited reversal of regional climate signals in overshoot scenarios. Env. Res: Clim..

[87] Schleussner C-F (2024). Overconfidence in climate overshoot. Nature.

[88] Liu L, Hauser M, Windisch M, Seneviratne S I (2025). Hysteresis and reversibility of agroecological droughts in response to carbon dioxide removal. Nat. Water.

[89] King A D, Lane T P, Henley B J, Brown J R (2020). Global and regional impacts differ between transient and equilibrium warmer worlds. Nat. Clim. Change.

[90] King A D, Borowiak A R, Brown J R, Frame D J, Harrington L J, Min S, Pendergrass A, Rugenstein M, Sniderman J M K, Stone D A (2021). Transient and Quasi–equilibrium climate states at 1.5$^\circ$C and 2$^\circ$C global warming. Earth’s Future.

[91] Dey S, Choudhary R K, Upadhyay A, Dash S K (2021). Aerosol-modulated heat stress in the present and future climate of India. Environ. Res. Lett..

[92] Van Oldenborgh G J, Wehner M F, Vautard R, Otto F E L, Seneviratne S I, Stott P A, Hegerl G C, Philip S Y, Kew S F (2022). Attributing and projecting heatwaves is hard: we can do better. Earth’s Future.

[93] Hetzer J (2024). Fire Weather Index for Europe from Downscaled and Bias-Corrected CMIP6 Model Outputs [Dataset].

